# Scapho-metacarpal dual mobility prosthesis for TMC-1 joint salvage: technical insights

**DOI:** 10.1007/s00402-025-05751-w

**Published:** 2025-01-15

**Authors:** Julia Glaser, Martin Aman, Thomas Krohn, Joris Duerinckx, Benjamin Panzram, Leila Harhaus

**Affiliations:** 1 BG Klinikum Unfallkrankenhaus Berlin, Department of Hand-, Replantation- and Microsurgery and Chair of Hand-, Replantation- and Microsurgery, Charité Universitätsmedizin Berlin, Berlin, Germany; 2 BG Trauma Center Ludwigshafen, Department of Hand, Plastic and Reconstructive Surgery, Burn Center, Plastic and Hand Surgery, University of Heidelberg, Ludwigshafen am Rhein, Germany; 3 St. Franziskus-Hospital, Ahlen, Germany Section Orthopedic, Trauma and Reconstructive Surgery, Ahlen, Ahlen, Germany; 4 Ziekenhuis Oost-Limburg, Genk, Departement of Orthopaedic Surgery and Traumatology, Genk, Belgium; 5 University Hospital Heidelberg, Section Upper Extremity, Orthopedic University Hospital Schlierbach, Heidelberg, Heidelberg, Germany

**Keywords:** Osteoarthritis of the trapeziometacarpal join, Rhizarthrosis, Dual mobily prosthesis for thumb TMC joint, Salvage strategy after failed trapeziectomy, Scaphometacarpal prosthesis

## Abstract

**Introduction:**

Rhizarthrosis, or osteoarthritis of the trapeziometacarpal joint, predominantly affects women over 50, with up to 30% experiencing some degree of arthritis in this joint. Traditional surgical approaches, such as trapeziectomy with ligament reconstruction, can result in some patients in persistent pain or limited functionality. TMC ball-in-socket arthroplasty, with a cup placed in the distal scaphoid, offers a promising alternative to traditional arthrodesis or resection-suspension arthroplasty.

**Materials and Methods:**

This study involved 11 patients with treated 13 hands who had persistent symptoms after previous TMC-1 surgeries. Procedures included a bilateral scaphometacarpal implantation in two cases. Main symptoms were pain, thumb shortening, and reduced grip strength. The Touch^®^ ball-in-socket prosthesis was used, with specific considerations for implant selection, surgical steps and customization based on the patient-specific case.

**Results:**

We included 11 patients with 13 thumbs, with a mean follow-up time of 16 months (range: 4–49 months). All patients showed significant improvements in thumb function. Grip strength, as measured by dynamometry, showed an average recovery to 80–90% of the contralateral side. Thumb opposition according to the Kapandji score averaged 9 out of 10. Radiographs demonstrated good osseointegration of the implants, with no signs of prosthetic loosening or dislocation. Complications included one case of persistent mild hypesthesia of the radial nerve’s superficial branch, which did not impair function, and one scaphoid fracture 4 weeks post-implantation during cast immobilization.

**Conclusion:**

The scapho-metacarpal dual mobility prosthesis is a feasible and effective option for patients with persistent TMC-1 symptoms after failed surgeries. It uniquely preserves both thumb mobility and length, unlike alternatives like arthrodesis and tendon suspensionplasty, which remain options if the prosthesis fails. Further research and long-term studies are necessary to determine the definitive role of this approach in complex TMC-1 cases.

## Introduction

Rhizarthrosis, or osteoarthritis of the trapeziometacarpal (TMC-1) joint, is a common condition, particularly amongst women over the age of 50. It is estimated that between 25 and 30% of individuals in this demographic suffer from some degree of TMC-1 arthritis. Furthermore, the prevalence of this condition increases significantly with age. The TMC-1 joint is essential for thumb mobility, particularly in activities requiring opposition and grip strength, making functional impairment in this joint highly debilitating. 

Traditional surgical interventions for advanced TMC-1 arthritis mainly involve trapeziectomy, with or without ligamentous reconstruction or tendon interposition, to relieve pain and restore function [1–3]. Despite generally favorable outcomes, a subset of patients experiences persistent pain, progressive proximalization of the thumb, or inadequate restoration of function. Revision surgeries are technically challenging and often result in suboptimal outcomes due to the altered biomechanics and soft tissue integrity [4, 5]. This underscores the need for innovative surgical techniques that can provide better functional outcomes in complex cases.

TMC ball-in-socket arthroplasty is gaining popularity as an alternative to trapeziectomy for painful TMC osteoarthritis [6–8]. Recently, its application in cases of failed trapeziectomy was proposed by placing the cup in the distal pole of the scaphoid instead of the trapezium. Clinical results of this technique are promising, but a detailed surgical technique was lacking [9]. The objective of the current report is to provide a detailed account of our own experience with this technique and a comprehensive description of the surgical procedure.

## Materials and methods

### Patient cohort

**Fig. 1:**
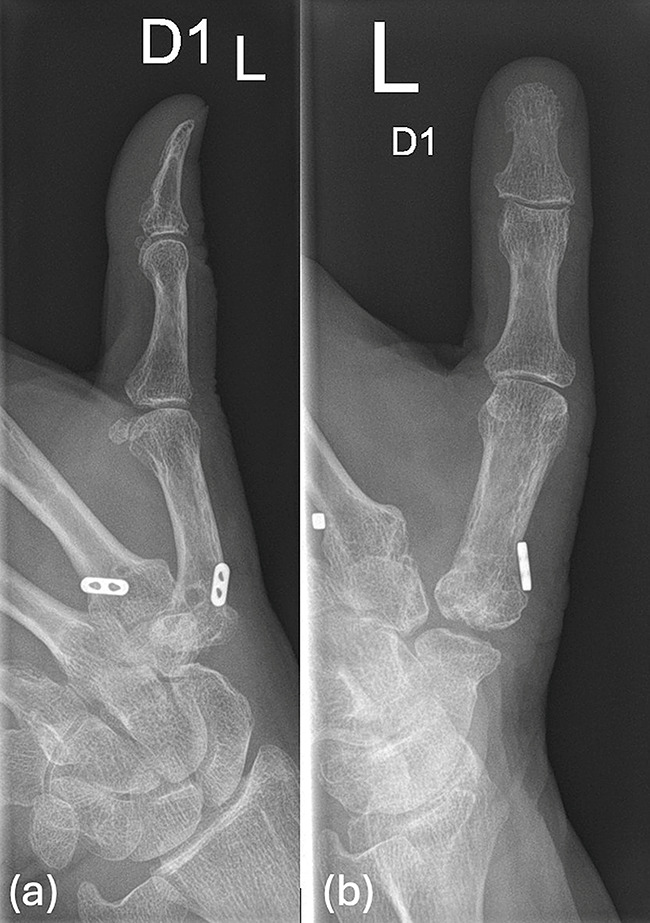
Radiographic views (**a**: lateral, **b**: frontal). Status after trapeziectomy and Tight Rope (Arthrex^®^) cable suspension. Significant subsidence of the thumb metacarpal in the resection space is evident. There is pronounced exostosis formation at the base of the first metacarpal bone. Distal scaphoid width was measured to be 9 mm

A total of thirteen hands were included in the analysis of eleven patients (nine females and four male) with ages ranging 52–80 years (mean 62 years), all of whom presented with persistent symptoms following previous surgeries for TMC-1 arthritis. Two of the 13 patients received bilateral scaphometacarpal implantation at intervals of two years each. Two of prior surgical interventions included trapeziectomy with suspensionplasty (Epping technique) and trapeziectomy with cable suspension with Tight Rope (Arthrex^®^) (Fig. 1).

Eight procedures were performed on the left hand and five on the right. The main symptoms were residual pain at the base of the thumb, thumb shortening and loss of grip strength.

The indication for scapho-metacarpal total joint arthroplasty was failure of conservative treatment and a demand of the patient to restore thumb mobility. The decision to opt for this surgery was made after informed consent of the patients, discussing the alternative surgical treatment options such as arthrodesis of the base of thumb and index metacarpal or revision suspension arthroplasty and the potential benefits and possible complications and the off-label use of a ball-in-socket implant as the cup is not intended for implantation in the scaphoid.

## Surgical technique

### Preoperative assessment and planning

All patients had painful grinding during axial compression and mobilization of the thumb. Preoperative radiographs demonstrated status after trapeziectomy with notable proximalization of the first metacarpal in all patients. For preoperative planning of the procedure, an X-ray of the thumb was performed in two views.

### Intraoperative considerations

The intraoperative approach focused on three key aspects: (1) the restoration of thumb length, (2) solid press-fit fixation of the prosthesis components and (3) correct orientation of the implant. The steps involved in this complex surgery are detailed below.

### Implant selection and customization

The Touch^®^ ball-in-socket prosthesis from Keri Medical is made primarily of stainless steel and titanium, with a highly cross-linked polyethylene (PE) liner for durability. It features a dual-mobility cup coated with titanium and hydroxyapatite (HA) to support bone integration [10]. Its double mobility articulation allows increased range of motion compared to the older single mobility systems and reduces the risk of dislocation.

An oversized stem (Touch Keri Medical^®^) was chosen to compensate for the shortening of the thumb caused by prior trapeziectomy. The scaphoid cup was selected based on the dimensions of the scaphoid, and adjustments were made intraoperatively to ensure optimal fit. The use of a conical or spherical cup was determined based on the morphology of the scaphoid, as previous studies have shown that both shapes provide good stability in different anatomical configurations. In most cases, the spherical cup is more advantageous than the conical one, as it allows for multiple adjustments to the positioning of the cup.

### Metacarpal stem

**Fig. 2:**
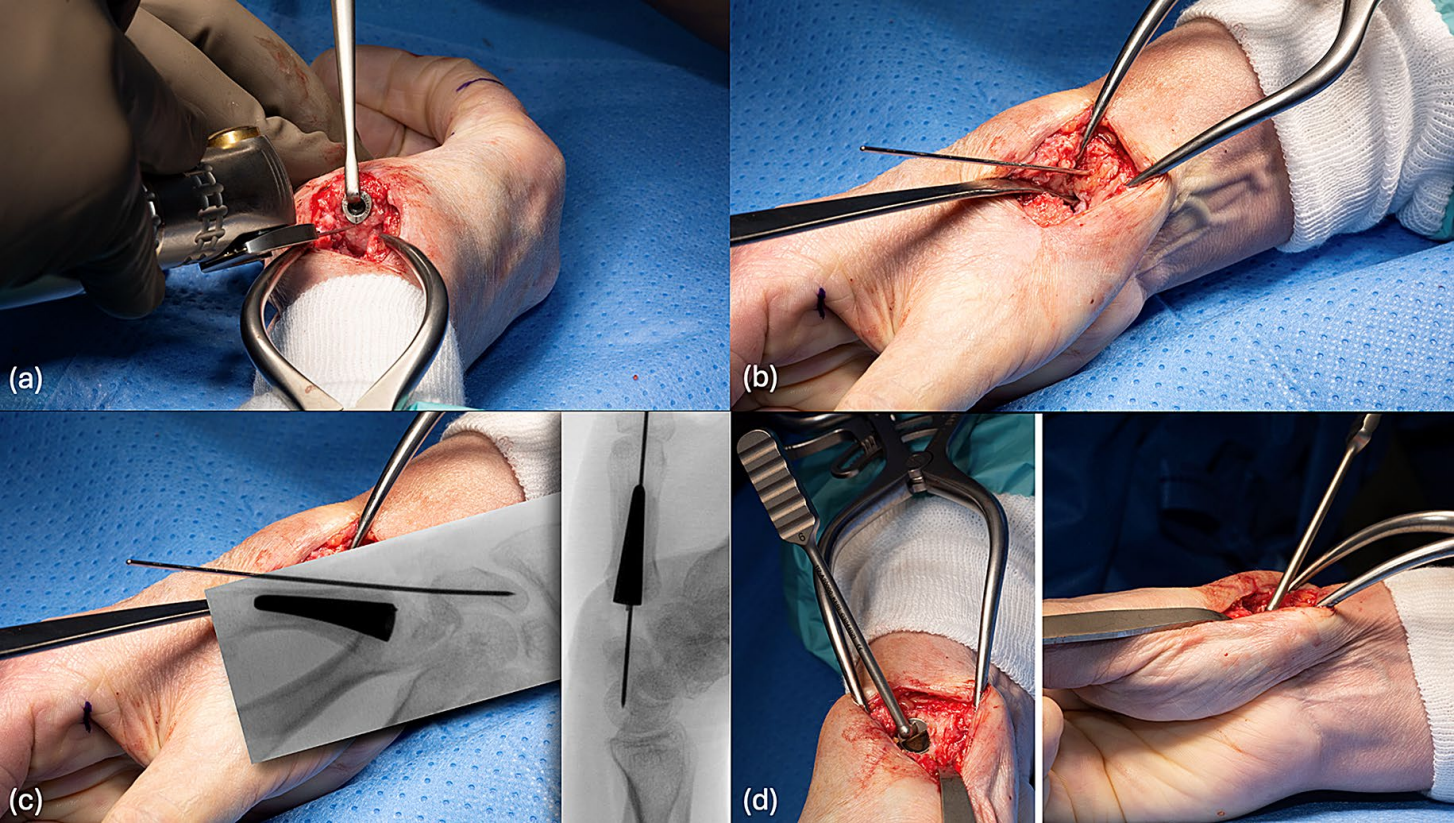
Intraoperative view of the successive surgical steps (**a**) positioning of the shaft in the thumb metacarpal. (**b**) Retrograde insertion of a K-wire in the distal pole of the scaphoid along its longitudinal axis. (**c**) Verification of K-wire position with fluoroscopy. (**d**) Assessment of press-fit stability using the cup template

The intramedullary canal of the thumb metacarpal was opened and prepared using cancellous bone impactors. No resection was made on the base of the metacarpal in order to allow a pressfit stabilization of the stem despite incomplete implantation and in order to maintain thumb length. The idea is to implant a larger stem than the size of the last intramedullary reamer used, so that the stem can be positioned proud from the metacarpal (Fig. 2). This technique allows to compensate for the loss of trapezium height. The average height of the normal trapezium bone is 16.07 ± 1.8 in male and 14.4 ± 1.3 mm in female patients [11]. This height is lost in these cases and must be compensated by the implant, rendering even the longest prosthesis neck insufficient in some cases to provide adequate soft tissue tension to stabilize the implant.

### Distal scaphoid cup

To broaden the access to the distal surface of the trapezium and to facilitate cup implantation, routinely resecting the lateral articular surface of the trapezoid is recommended. A K-wire was used to secure alignment during the scaphoid preparation, and the scaphoid was drilled using the attached guide to ensure precision (Fig. 2).

Fluoroscopy was used to evaluate the position of the implant during surgery (Fig. 3). The correct position of the cup in fluoroscopy was verified by ensuring that the prosthetic cup in the trapezium was placed parallel to the distal articular surface of the scaphoid to guarantee a full physiological thumb motion [12, 13]. The socket of the prosthesis must be fully supported by the scaphoid.

### Radiographic and fluoroscopic guidance

**Fig. 3:**
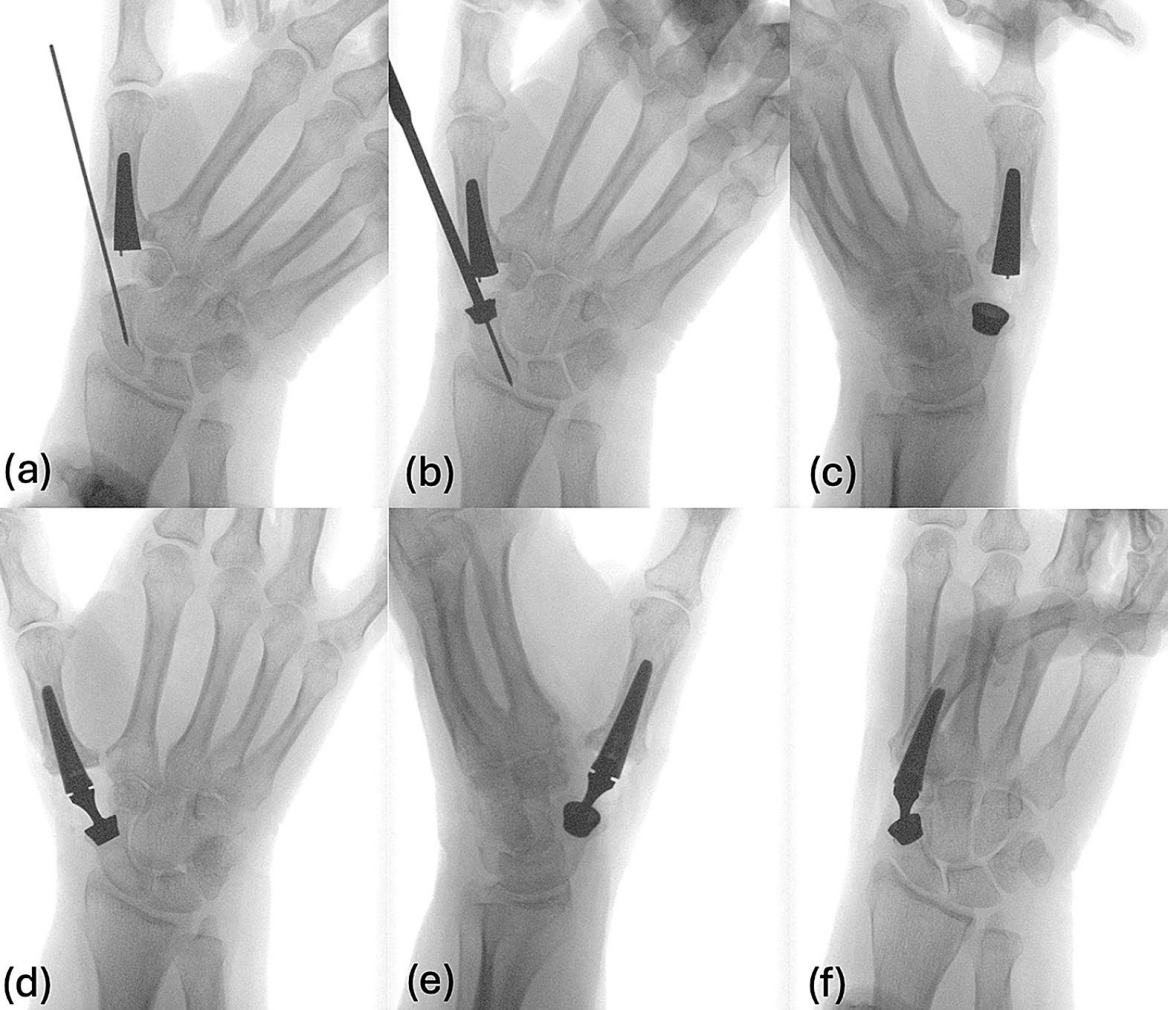
Individual surgical steps (**a**) shaft introduced in the first metacarpal bone. K-wire placed in the scaphoid. (**b**) pre-drilling of the scaphoid. (**c**) positioning of a conical cup in the scaphoid. (**d**) insertion of the neck. (**e**) X-ray in a second plane. (**f**) stress-test of the prosthesis with maximal opposition

### Intraoperative stability testing

After implantation, intraoperative testing was performed to assess range of motion and of the prosthesis longitudinal laxity of 2–3 mm was considered appropriate.

Intraoperative stress testing helped confirm the stability of the prosthesis under dynamic conditions, reducing the risk of dislocation or suboptimal functional outcomes. Here, potential prosthesis dislocation is examined using telescoping with axial traction on the thumb. To achieve a stable and functional positioning of the prosthesis, it is essential to assess wrist extension and flexion, as well as ulnar and radial deviation, given that the scaphoid is part of the proximal carpal row and contributes to the radiocarpal joint. These movements should be tested, considering all degrees of freedom to prevent dislocation. A test of the prosthesis is conducted to ascertain its stability under load, employing a push-and-pull motion of the thumb. The hitchhiker movement (thumb extension) is also assessed to check for proper range and ease of motion. Kapandji testing is performed, which measures the thumb’s ability to reach towards the fingers, evaluating opposition and flexibility. Retropulsion (ability to extend the thumb backward) is another component that needs to be examined for full thumb mobility. Finally, adduction in the first web space is tested to confirm the thumb’s ability to move inward, ensuring functional grip and hand strength.

This comprehensive intraoperative testing ensures the prosthesis is properly positioned and capable of supporting the full range of thumb movements.

Existing capsule and scar tissue is mobilized during the closure of the joint to achieve joint closure. However, this is often not as easily achievable in the context of a revision surgery as in the initial procedure. Reinforcement with mesh or tendon tissue has not been performed. A tight capsule closure with (scar) tissue is desirable.

### Postoperative care

Patients were immobilized using a thumb splint for the first six weeks postoperatively, ensuring that the wrist and IP-joint remained free. Early mobilization exercises were introduced under physiotherapy supervision from the third postoperative week, focusing on circular movements of the thumb and ensuring smooth articulation between the metacarpal and scaphoid bones.

### Progressive loading and strengthening

After six weeks patients began progressive strengthening exercises. The use of circular motion and grip-strengthening protocols was encouraged.

## Results

**Fig. 4:**
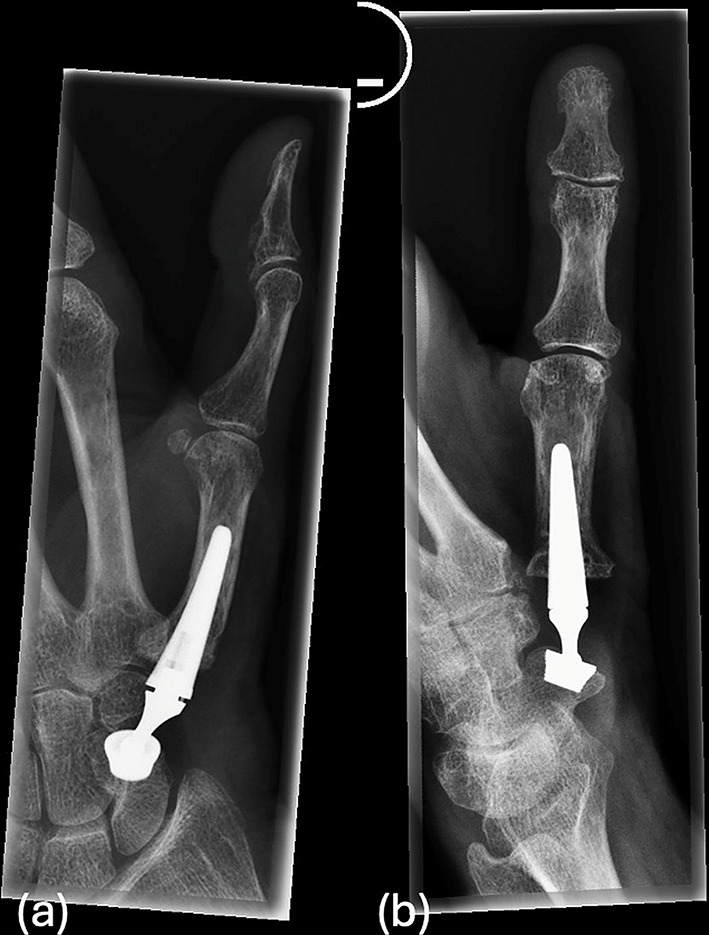
Six weeks postoperatively, the implanted Touch prosthesis (Keri Medical, 9 mm conical cup in the scaphoid, 15° neck size M, shaft size 1) is positioned between the scaphoid and the first metacarpal bone. No signs of prosthetic loosening are observed

**Fig. 5:**
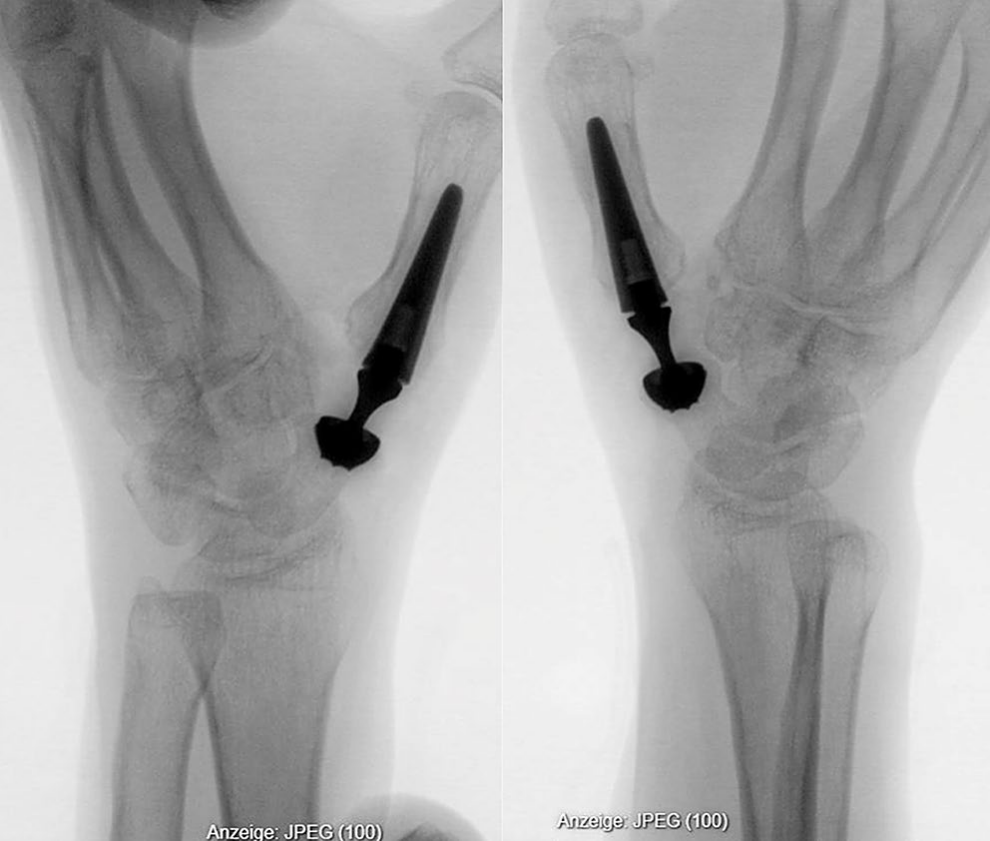
Intraoperative fluoroscopy in two planes to verify the positioning after implantation of a scaphometacarpal touch prosthesis with a spherical cup

**Fig. 6:**
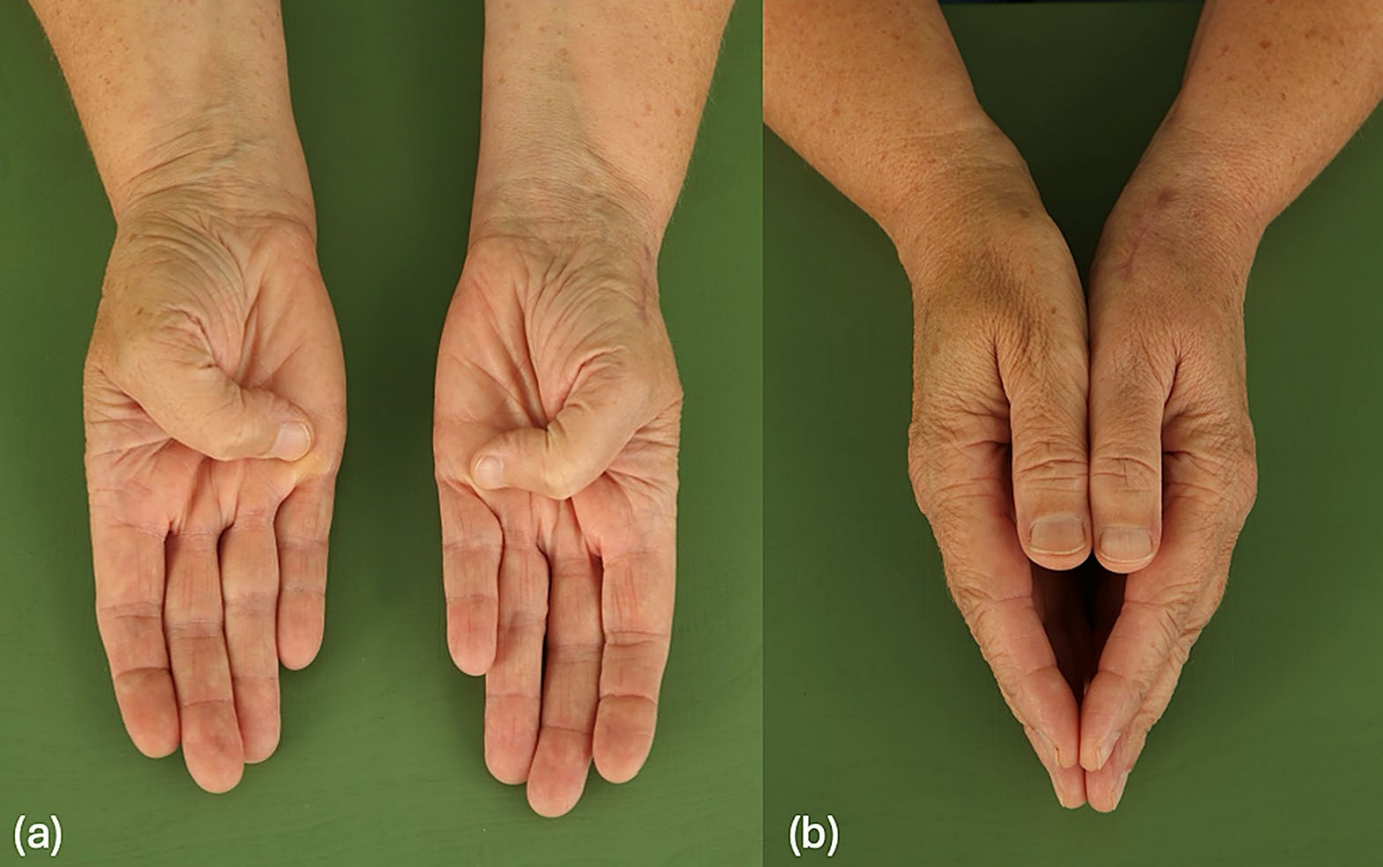
Clinical result 12 weeks postoperatively. (**a**) Full opposition Kapandji 10. and surgical scar healing. (**b**) The length of the thumb is comparable to the other side

We included 11 patients with 13 thumbs, with a mean follow-up time of 16 months (range 4-49months). All patients showed significant improvements in thumb function. Grip strength, as measured by dynamometry, showed an average recovery to 80–90% of the contralateral side. Two patients achieved a grip force greater than 100% compared to the other side during follow-up, likely explained by the operation on the dominant hand (Table 1). Radiographs demonstrated good osseointegration of the implants, and no signs of prosthetic loosening or dislocation (Figs. 4 and 5). Thumb opposition according to Kapandji score averaged 9 out of 10 (Fig. 6). Cases 9 and 10, as well as Cases 11 and 12, involved bilateral implantation of the prosthesis in a single patient.

**Table 1:** Key demographics and surgical history of the patient cohort. Satisfaction: 1/5 = low satisfaction, 5/5 high satisfaction

Pat.	Sex	Age	Follow-up (months)	Side	Prior Surgical Procedures	Grip force compared to other side (%)	Kapandji Score	Satisfaction (1–5)
1	f	57	17	R	Epping + Tight Rope^®^	90%	9	5
2	f	62	16	L	Epping	95%	9	5
3	f	52	11	R	Epping	25%	10	3
4	f	59	7	R	Epping	85%	8	4
5	f	61	5	L	Epping	85%	9	5
6	m	63	4	L	Epping	90%	9	5
7	f	58	4	L	Epping + Tight Rope^®^	x	10	4
8	m	61	29	L	Epping + Tight Rope^®^	x	x	x
9	f	68	36	L	Epping	> 100%	9	5
10	f	70	10	R	Epping	70%	9	5
11	m	58	49	R	Epping	x	8	5
12	m	61	16	L	Epping	x	8	5
13	f	80	6	L	Epping	> 100%	10	5

Complications included one case of mild hypesthesia of the superficial branch of the radial nerve, which persisted in one patient but did not impair function, and one case of postoperative failure due to a fracture of the scaphoid that occurred 4 weeks after implantation during cast immobilization (patient 8). The cup was presumably placed too far laterally into the distal scaphoid. The prosthesis was completely removed. Hindersome MCP1 hyperextension due to thumb shortening was treated with MCP1 arthrodesis.

## Discussion

The use of a scapho-metacarpal dual mobility prosthesis for TMC-1 joint salvage after failed trapeziectomy represents an innovative yet technically challenging approach. This case series demonstrates its potential for restoring thumb length and function, often compromised in patients who have undergone multiple surgeries. Here, we explore the technical aspects of the method, review alternative approaches for TMC-1 arthritis revision surgery, and evaluate the current literature to contextualize the outcomes in our patient cohort.

### Dual mobility prosthesis in TMC-1 joint salvage

The dual mobility prosthesis offers several advantages over traditional techniques like arthrodesis or revision surgery, such as resuspension or interposition. By preserving thumb mobility, this prosthesis addresses a primary functional deficit in patients post-trapeziectomy, particularly those with significant proximalization and shortening of the thumb. One of the key benefits is the ability to restore thumb length by using an oversized stem, which directly correlates with improved grip strength, opposition, and overall hand function [9].

Another advantage of the dual mobility prosthesis is early mobilization. Most patients can start physiotherapy within weeks after surgery, significantly reducing the recovery time compared to fusion techniques [7]. In our series, patients reported high satisfaction and demonstrated rapid functional improvement. Radiological evidence of good osseointegration supports the long-term viability of this approach, although longer follow-up is needed to fully evaluate its durability.

However, implantation of the Touch^®^ prosthesis demands meticulous attention to detail. Proper component positioning is crucial to avoid postoperative complications such as dislocation or loosening of the prosthesis. In our series, intraoperative fluoroscopic guidance was essential to ensure accurate alignment, corroborating findings from other studies [14]. Furthermore, intraoperative stress testing helped confirm the stability of the prosthesis under dynamic conditions, reducing the risk of dislocation or suboptimal functional outcomes.

### Alternative methods for TMC-1 joint salvage

While the dual mobility prosthesis offers clear advantages, it is not the only option for patients with failed TMC-1 surgeries. Several alternative methods are available, each with its own benefits and limitations.

Arthrodesis (Joint Fusion): Arthrodesis, or fusion of the first metacarpal to the scaphoid or second metacarpal, is often considered the gold standard for patients with multiple failed surgeries [15]. By eliminating joint movement, arthrodesis can offer significant pain relief and restore stability, which is especially valuable for patients with high physical demands. However, the major drawback of arthrodesis is the loss of thumb mobility, which severely limits functions like opposition and grip. While it can provide good pain control, studies show that the loss of function makes it less desirable for patients who require thumb mobility for fine motor tasks or daily activities [1].

Resection-Suspension arthroplasty: Techniques such as the Epping or Lundborg procedures use autologous tendon grafts to stabilize the first metacarpal following trapeziectomy [15]. These methods can provide short-term and even long-term pain relief and preserve thumb length. However, in revision surgeries, outcomes are less predictable, and patients often experience persistent pain or loss of thumb length, as seen in our cohort. Tendon elongation or failure over time remains a significant concern for the long-term success of these techniques [8].

Tight Rope Fixation: The Tight Rope^®^a system (Arthrex^®^) offers a minimally invasive solution for stabilizing the first and second metacarpals after trapeziectomy. Although it provides immediate stability, our experience, supported by the literature, shows that the Tight Rope system can fail in patients with severe proximalization or significant thumb shortening [9]. Several studies report mixed results, with some patients experiencing persistent pain and inadequate functional restoration after Tight Rope fixation [7].

Implant Arthroplasty with Non-Dual Mobility Prostheses: Various prosthetic designs, such as surface replacement arthroplasty or ball-and-socket implants, have been developed for treating TMC-1 arthritis [1]. While these implants can offer pain relief, they have higher rates of dislocation and loosening compared to dual mobility prostheses. Additionally, these designs typically have a limited range of motion, making them less suitable for patients needing full thumb opposition. Comparative studies consistently show that dual mobility prostheses outperform these designs in terms of motion range and dislocation rates [7].

### Comparative literature and long-term outcomes

The literature on dual mobility prostheses for primary TMC-1 arthritis is growing, though data on its use in revision surgeries remain limited. Studies primarily focus on primary settings, where dual mobility prostheses show significant benefits, including better preservation of thumb length, reduced pain, and faster recovery [9]. A lower complication rate compared to trapeziectomy alone has also been documented, suggesting that dual mobility prostheses may be advantageous in revision cases, but long-term data are still needed.

One common concern with dual mobility prostheses is the risk of dislocation, particularly in revision cases. Some studies report dislocation rates in patients undergoing primary and revision surgery, though our findings align with the literature suggesting that careful surgical technique and intraoperative testing can minimize this risk [14]. Radiological confirmation in multiple planes is essential to ensure proper positioning and reduce the likelihood of prosthesis failure.

Wear and tear of the prosthetic components is another concern, particularly in younger or more active patients. Long-term studies are necessary to quantify the extent of wear and identify complications such as osteolysis or loosening. However, early results suggest that dual mobility prostheses may offer longer-lasting outcomes compared to other designs, especially in revision surgeries [7].

### Limitations and future directions

A key limitation of our case series is the small sample size and relatively short follow-up period. While our results are promising in terms of patient satisfaction and functional outcomes, larger studies with longer follow-ups are needed to confirm these findings. Additionally, future research should focus on comparing the dual mobility prosthesis with other revision techniques, such as arthrodesis and suspensionplasty, in terms of both short- and long-term results.

Another area for future research is the development of patient-specific implants. While using an oversized stem provided adequate restoration of thumb length in our cases, custom implants designed to match individual anatomy may improve outcomes. Advances in 3D printing and implant design could lead to more precise and successful revision surgeries for TMC-1 arthritis.

## Conclusion

The scapho-metacarpal dual mobility prosthesis offers a technically feasible and effective option for patients with persistent TMC-1 joint symptoms following failed surgeries. While alternative methods such as arthrodesis and tendon suspensionplasty are available (and still are in case of a possible failure of the prosthesis), the dual mobility prosthesis uniquely preserves both thumb mobility and length. Further research and long-term studies are required to establish its definitive role in complex TMC-1 cases.

## Data Availability

No datasets were generated or analysed during the current study.
