# Correction: Scapho-metacarpal dual mobility prosthesis for TMC-1 joint salvage: technical insights

**DOI:** 10.1007/s00402-025-06050-0

**Published:** 2025-10-17

**Authors:** Julia Glaser, Martin Aman, Thomas Krohn, Joris Duerinckx, Benjamin Panzram, Leila Harhaus

**Affiliations:** 1 BG Klinikum Unfallkrankenhaus Berlin, Department of Hand-, Replantation- and Microsurgery and Chair of Hand-, Replantation- and Microsurgery, Charité Universitätsmedizin Berlin, Berlin, Germany; 2 BG Trauma Center Ludwigshafen, Department of Hand, Plastic and Reconstructive Surgery, Burn Center, Plastic and Hand Surgery, University of Heidelberg, Ludwigshafen am Rhein, Germany; 3 St. Franziskus-Hospital, Ahlen, Germany Section Orthopedic, Trauma and Reconstructive Surgery, Ahlen, Ahlen, Germany; 4 Ziekenhuis Oost-Limburg, Genk, Departement of Orthopaedic Surgery and Traumatology, Genk, Belgium; 5 University Hospital Heidelberg, Section Upper Extremity, Orthopedic University Hospital Schlierbach, Heidelberg, Heidelberg, Germany

**Correction: Archives of Orthopaedic and Trauma Surgery 145:128 (2025)** 10.1007/s00402-025-05751-w.

The article Scapho-metacarpal dual mobility prosthesis for TMC-1 joint salvage: technical insights, written by Julia Glaser, Martin Aman, Thomas Krohn, Joris Duerinckx, Benjamin Panzram and Leila Harhaus was originally published under exclusive license to The Author(s), under exclusive licence to Springer-Verlag GmbH Germany, part of Springer Nature 2025. As a result of the subsequent decision to publish the article under the open access model, the article’s copyright notice was changed on 18 August 2025 to © The Author(s) 2025 and This article is licensed under a Creative Commons Attribution 4.0 International License, which permits use, sharing, adaptation, distribution and reproduction in any medium or format, as long as you give appropriate credit to the original author(s) and the source, provide a link to the Creative Commons licence, and indicate if changes were made. The images or other third party material in this article are included in the article’s Creative Commons licence, unless indicated otherwise in a credit line to the material. If material is not included in the article’s Creative Commons licence and your intended use is not permitted by statutory regulation or exceeds the permitted use, you will need to obtain permission directly from the copyright holder. To view a copy of this licence, visit http://creativecommons.org/licenses/by/4.0/.

Open Access funded by: Open access funding enabled and organized by Projekt DEAL.

The original article has been updated.

